# Auditory steady-state responses in primary and non-primary regions of the auditory cortex in neonatal ventral hippocampal lesion rats

**DOI:** 10.1371/journal.pone.0192103

**Published:** 2018-02-07

**Authors:** Sibin Li, Lanlan Ma, Yuchen Wang, Xuejiao Wang, Yingzhuo Li, Ling Qin

**Affiliations:** Department of Physiology, College of Basic Medical Science, China Medical University, Shenyang, Liaoning Province, P. R. China; Bilkent University, TURKEY

## Abstract

Auditory steady-state responses (ASSRs) represent the electrophysiological activity of the auditory nervous system in response to a periodic acoustic stimulus. Spectrogram analysis can reveal the frequency and phase information entrained in ASSRs. Clinically, the ASSR is used to detect abnormalities in electroencephalographs obtained from schizophrenia patients, who show reduced power and phase locking of ASSRs. The neonatal ventral hippocampal lesion (NVHL) rat is a widely used model to investigate the neurodevelopmental mechanisms of schizophrenia. It has been established that NVHL rats exhibit several schizophrenia-like behavioral and molecular abnormalities. However, no clear abnormalities in ASSRs have been reported to date. The present study compared ASSRs of adult NVHL and sham-operated rats. We inserted microelectrodes into the primary auditory cortex (A1) or posterior auditory field (PAF) and recorded the local field potential (LFP) in response to 40- and 80-Hz click train stimuli. Spectrogram analysis was performed to obtain the mean trial power (MTP) and phase-locking factor (PLF) of the click train-evoked LFPs. We found that in the control animals, A1 showed a stronger MTP and PLF of ASSR than PAF, and NVHL operation mainly impaired the ASSR in PAF. Analysis of spike activity also indicated that NVHL operation extended the duration of tone-evoked responses in PAF neurons. Our results reveal, for the first time, that NVHL may distinctly influence the neural activities of primary and non-primary fields of the auditory cortex.

## Introduction

The disruption of neural synchronization and information integration is considered a key pathological characteristic of schizophrenia [1]. Auditory steady-state responses (ASSRs) are neural activities of the auditory nerve system in response to periodic acoustic stimuli that can be used to test neural synchronization [2, 3]. ASSRs in patients with schizophrenia are typically reduced in power or phase synchronization in response to a 40-Hz stimulation. This has been observed in first episode schizophrenia patients, in adolescents with a diagnosis of a psychotic disorder, and in first-degree relatives of schizophrenia patients [4–8]. ASSR deficits in the 40-Hz range suggest the function of auditory cortex (AC) is disturbed in schizophrenia [9]. To data, there have been several lines of evidence supporting that AC is damaged in the schizophrenia patients. First, individuals with schizophrenia commonly show auditory symptoms including auditory hallucinations. Second, magnetic resonance imaging studies have attributed the auditory symptoms in schizophrenia to the altered activation of the AC [10–13]. Third, loss of dendritic spines and alterations of synaptic signaling have been observed in the AC of schizophrenia patients [14–15]. Thus, the AC is a worthy subject of schizophrenia research.

The neonatal ventral hippocampal lesion (NVHL) rat is a widely used neurodevelopmental animal model of schizophrenia [16]. The establishment of this model was inspired by the evidence that schizophrenia patients show a lateral ventricular enlargement and hippocampal changes. NVHL triggers numerous behavioral, molecular, and physiological changes in schizophrenia patients. Although the direct projection from the hippocampus to the AC is not so dense, there probably are some indirect projections bypassing the amygdala and the auditory thalamus [17]. This possibility is implied by the fact that NVHL rats show abnormal behavioral responses to acoustic stimuli [18]. NVHL rats also show several brain electrophysiological abnormalities in the auditory evoked potential and sensory gating, similar to those observed in schizophrenia [19–21]. However, previous studies on NVHL rats did not reveal ASSR deficits common to schizophrenia patients [22, 23]. This may be attributed to the methodology of ASSR recording used in most previous studies, involving electroencephalographs (EEGs) obtained from the brain surface. Because the rat brain has a number of small functional divisions, surface EEG recordings cannot differentiate between the detailed neural activities in different cortical regions. Therefore, it is necessary to use a more accurate method to examine ASSRs in the cortex, particularly in the AC, which is considered an area of ASSR origin. Anatomical and electrophysiological mapping studies have confirmed that the AC consists of primary and non-primary regions, which receive different thalamocortical projections and show different neural responses to acoustic stimuli. Structural and functional differences between the primary and non-primary AC might result in different ASSR characteristics and sensitivities to the NVHL. To examine this, we recorded local field potentials (LFPs) through microelectrodes in rat AC to investigate ASSR across the primary AC (A1) and posterior auditory field (PAF), representative primary and non-primary regions, respectively. Our results demonstrate that the power and phase-locking of LFP were significantly decreased in the PAF of NVHL rats. The parameters of ASSR in the A1 were less affected by NVHL. These data suggest that neural activity in the non-primary AC region correlates with abnormalities in ASSRs seen in schizophrenia.

## Materials and methods

### Animals

All experimental protocols were approved by the China Medical University Animal Care and Use Committee (permit number: 2014195) and were in strict accordance with the National Institute of Health Guide for the Care and Use of Laboratory Animals (NIH Publications No. 80–23) revised in 1996. All surgery and electrophysiological experiments were conducted under anesthesia with maximum effort taken to reduce animal suffering. Pregnant Sprague—Dawley rats were obtained from our animal facilities and housed in individual cages (50 × 35 × 20 cm) with a 12-hour light-dark cycle. The animals were provided with a chow diet (Global 18% protein rodent diet; Maohua Biology, Xinmin, Liaoning, China) and water *ad libitum*. During the time leading up to parturition, care was taken to closely monitor but not overly disturb the animals, at 12-hour intervals. The animal health and well-being was assessed by monitoring for signs of distress, including trembling, vocalization, changes in normal activity, and changes in urine/fecal mass. Normal pre-labor and nesting behavior were also monitored.

### Neonatal ventral hippocampal lesion

Seven-day-old male pups were randomly assigned to either the sham or NVHL groups. The pups were anesthetized via hypothermia by placing the animal at a 0°C chamber in a refrigerator for 10–12 min. The anesthesia was considered complete, when the distal limbs were no longer pink and no spontaneous limb movement was observed. Thereafter, the pups were fixed on a custom-made platform attached to a stereotaxic apparatus (SN-2N, Narishige, Japan). An incision was made into the skin to expose the cranium, and two small holes (diameter: approximately 1 mm) were drilled into the skull bone. A steel tube was implanted bilaterally into the ventral hippocampus. The coordinates used were AP −3.0 mm, ML ±3.5 mm, and DV −5.0 mm. Ibotenic acid (3 μg in 0.3 μL, Sigma, St Louis, MO, USA), dissolved in 0.15 M phosphate buffer saline (PBS, pH = 7.4) or vehicle (sham), was infused into the ventral hippocampus through the implanted tube at a flow rate of 0.1 μL/min.

Rats were weaned on postnatal day 25 and moved to standard plastic cages (two per cage) on postnatal day 49. To reduce stress, rats were handled daily until the commencement of the electrophysiological recording experiments. Three days before the experiments, rats were moved again, in order to be housed separately in single-occupancy Plexiglas cages.

### Prepulse inhibition test

On postnatal day 65, we used the prepulse inhibition of the acoustic startle response (PPI) paradigm to examine whether NVHL rats showed schizophrenia-relevant behavioral deficits [24]. The PPI test is based on the phenomenon that a weak sound presented 30–500 ms before a startling sound reduces the amplitude of the startle response. One session of the PPI test consisted of seven stimuli delivered in a pseudorandom order: 1) pulse alone (100 dB sound pressure level (SPL) white noise, 20 ms duration); 2) control (no stimulus); 3) and 4) prepulse alone (72 or 68 dB, pure tone, 10 kHz, 20 ms duration); 5), 6), and 7) prepulse (72, 68, or 64 dB) each followed by a pulse with an inter-stimulus interval of 100 ms. A total of 10 presentations of each type was given with an inter-trial interval randomized between 20 and 30 s. Background noise intensity during the whole experiment was 60 dB SPL. The PPI was calculated according to the formula 100 − 100% × (PPx/PA), in which PPx is the startle reactivity of the 10 PPI trials (separate for each individual prepulse intensity) and PA is the startle reactivity of the pulse alone trials. The average PPI response over the three prepulse intensities was analyzed.

### Electrophysiological recordings

#### Surgical preparation

Upon reaching 350 ± 50 g of weight (around postnatal day 70), the rats underwent surgery for performing electrophysiological recordings. The animals were anesthetized by an intraperitoneal injection of urethane (1.5 g/kg) and supplementary doses (0.5 g/kg) were administered as needed. Dexamethasone (0.25 mg/kg) was administered every 4 h to prevent brain edema. Atropine sulfate (0.1 mg/kg) was used to reduce the volume and viscosity of bronchial secretions. An electric blanket was used to maintain a rectal temperature of 37°C. A custom-made metal block was implanted onto the rat’s skull to hold the head during recording experiments. A craniotomy was performed above the area of the AC, for which the coordinates used were AP −3.0 to −7.0 mm, ML 3 to 5 mm [25].

#### Recording procedure

**Fig 1:**
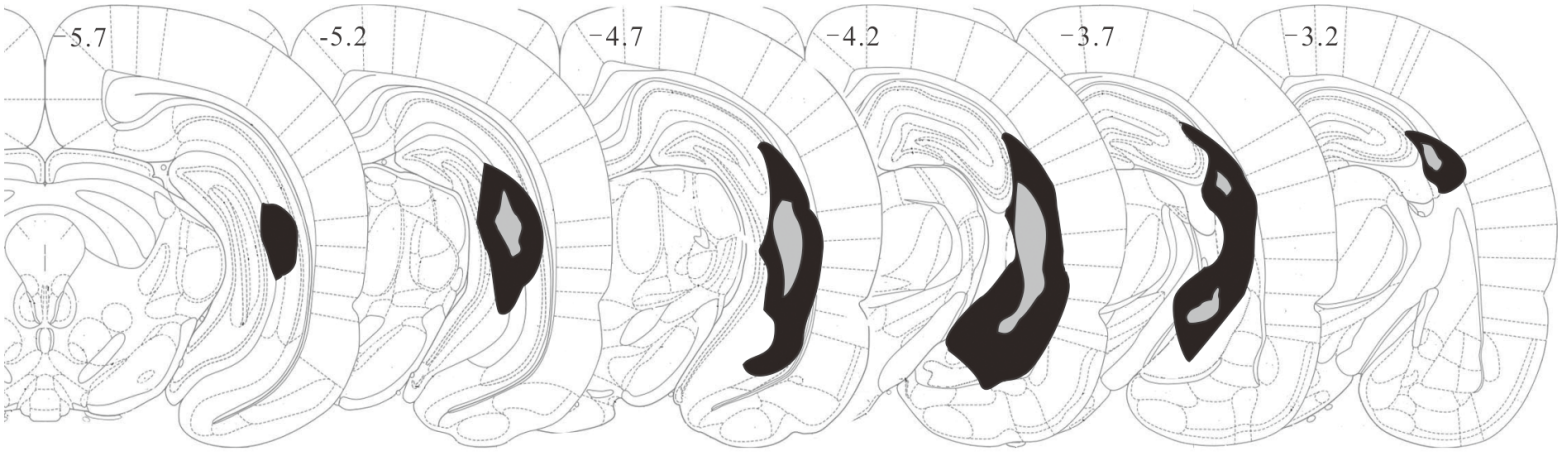
Schematic drawing of coronal sections illustrating the lesions of NVHL rats, as determined by the examination of Nissl-stained sections. Dark regions show the largest and grey regions show the smallest extent of damage across the 6 NVHL rats (on either side). Numbers indicate the distance in mm from the bregma according to the rat brain atlas.

Electrophysiological recordings were conducted in an electrically shielded, soundproof box soon after the surgery. During the recordings, the ear bars were removed, and the rat’s head was held via the metal block implanted onto the skull. A single epoxylite-insulated tungsten microelectrode (#575500, A-M systems, WA, USA) was positioned orthogonal to the brain surface. A motor-driven manipulator (SM-20, Narishige, Tokyo, Japan) was used to insert the microelectrode 400–600 μm into the brain, corresponding to the thalamorecipient layers III-IV. The signal was amplified with a differential amplifier (RA16PA, TDT, Alachua, FL, USA). This analog signal was then digitized, amplified, and filtered (1 and 300 Hz) using the RZ2 processor (TDT) to obtain LFPs. In some cases, neural discharge signals (spike activity) could also be recorded from the electrode. To obtain a clear spike activity signal, the electrode output was filtered by a 0.3–5 kHz bandpass filter, and spikes were detected online by the threshold crossing and waveform templates. Data of the LFP waveforms and spike times were stored on a hard disk for offline analysis. After completing the recording at one site, the electrode was withdrawn and moved horizontally 0.5–1 mm in a randomized direction, avoiding vessel branches, to record at other sites. The entire recording session lasted 24–36 h, during which 30–50 sites were sampled, evenly covering the A1 and PAF of one hemisphere (Fig 1).

### Acoustic stimuli

Sound stimuli were presented via a free-field speaker (K701, AKG, Austria) placed 50 cm away from the contralateral ear. The sounds used to elicit and isolate a neuronal response were a set of pure tones (160 ms duration, 5 ms linear rise/fall time) presented in a random sequence of different frequencies, ranging from 128 Hz to 32 kHz on a logarithmic scale (0.06 octave frequency interval and 1.5–3.0 s inter-stimulus interval), at 70 dB SPL. ASSRs were measured using 500-ms click trains with 40- or 80-Hz repetition rate. The duration of each click was 1 ms with an amplitude of 70 dB SPL. One recording block consisted of 120 trials (60 trials of 40- and 80-Hz click trains each), which were randomly interleaved in inter-train intervals of 2–4 s.

### Data analysis

To analyze tone-evoked responses, we set a time window from the stimulus onset to 50 ms after stimulus cessation (0–550 ms). The maximum deflection during this time window was the LFP amplitude. The best frequency (BF) of each recording site was estimated as the tonal frequency evoking the maximum LFP amplitude. Click-evoked LFPs were filtered using a digital bandpass filter with a lower and higher cut-off frequency adjusted to 5 Hz below and above the repetition rate of click train stimuli. The average evoked LFP power, mean trial power (MTP), and phase-locking factor (PLF) were analyzed using a wavelet-based analysis algorithm [26] implemented through custom-written MATLAB scripts. Wavelet analysis provides a dynamic tradeoff between the resolutions of time and frequency domain, by using small temporal widths for high frequencies and large temporal widths for low frequencies. Contrastingly, the standard Fourier transformation uses a fixed temporal width for all frequencies. To obtain MTP, we first computed the power of each individual trial LFP and then subtracted the mean power during the baseline period (from −500 to 0 ms, relative to stimulus onset) from the power of each trial. The PFL measures the synchronization of LFP phases across individual trials at particular frequencies and time intervals.

Spike activities driven by pure tone stimuli were aligned with stimulus onset, to construct a raster plot for each tone frequency. The peri-stimulus time histogram (PSTH), generated by counting the spikes across the 125 trials of different frequencies, was computed in 1 ms bin width and smoothed by the Gaussian function with 5 ms standard deviation (SD). The threshold to identify a significant response was set as the mean background spike rate (taken from 0.5 s preceding sound onset) ± 3 SD. Response duration was estimated by counting the PSTH supra-threshold time bins.

### Calibration of recording sites and histology

After completing the recording experiments, the AC surface was photographed with a digital camera, and the photos were edited on a computer screen. The absolute scale and position of the explored brain area was estimated with respect to the bregma, by using reference points, which had been previously marked on the temporal bone. A coordinate grid was added onto the photographs to guide and mark the sites of the recording electrode.

Following completion of imaging of the cortex, the rats were deeply anesthetized and their brain fixed by 4% paraformaldehyde perfusion through the heart. The brain tissue from 3.30–5.80 mm posterior to the bregma was cut into 40 μm thick coronal slices and stained with thionin. An experimenter blinded to the electrophysiological results was responsible for evaluating the extent of the lesions by examining the thionin-stained slices under the microscope. Sham-operated rats with damage to the hippocampus and NVHL rats without successful hippocampal lesions were excluded from further analysis. Fig 1 shows the histological results of the 6 NVHL rats used in the study with the greatest (black) and least (grey) extent of lesion.

## Results

### PPI of the acoustic startle response is disrupted in NVHL rats

**Fig 2:**
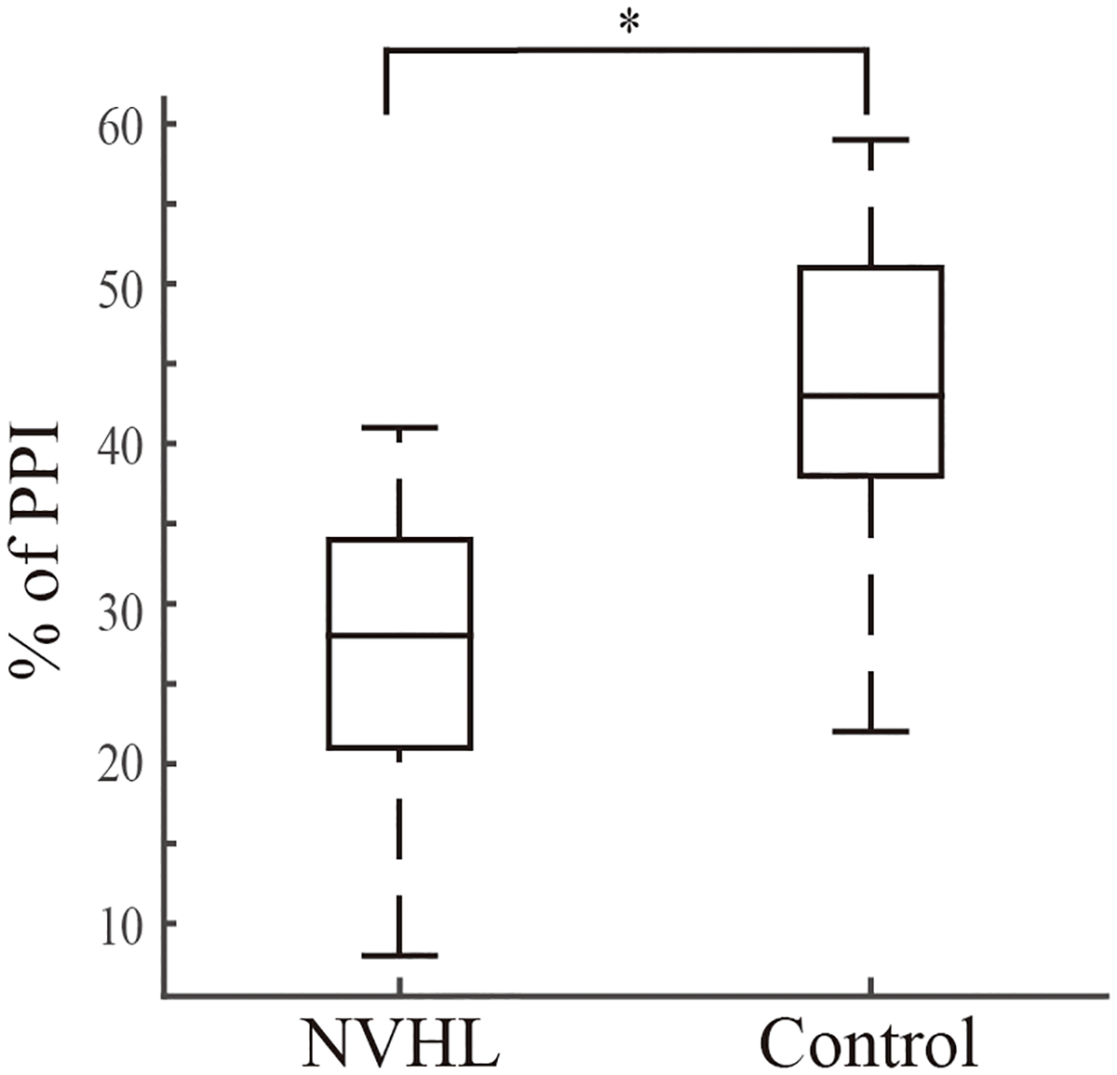
PPI in the NVHL and sham rats. NVHL rats showed a smaller PPI than sham rats. The inner line and edges of the box represent the median, the 25, and 75 percentiles respectively, while the whiskers show the range as mean ± 3 SD. *p < 0.05, *t* test.

The 6 NVHL rats used in this study showed a significant decrease in PPI compared with the 6 sham rats (Fig 2; p = 0.04, *t* test; Cohen’s d effect size = 1.33). This result indicates that our NVHL method successfully resulted in a schizophrenia-like behavioral phenotype in the rats.

### Identification of A1 and PAF

**Fig 3:**
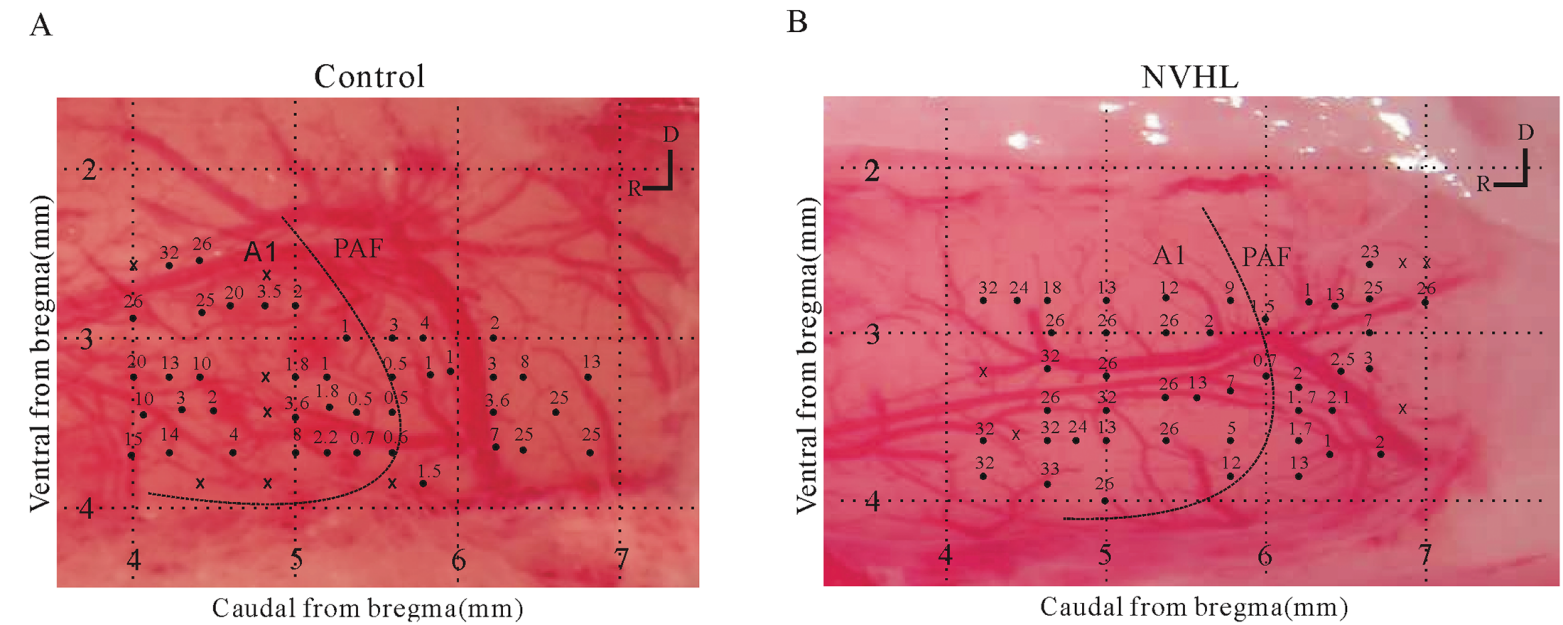
(A) Sample case with 49 LFP recording sites in the A1 and PAF of a representative sham rat. Numbers show the BF in kHz of each recording site. Crosses represent a site that had no response to pure tone stimuli. The boundary between the A1 and PAF (dashed curve) is estimated on the basis of the reversal of the BF gradient. Along the dorsocaudal direction in the A1, there is a high-to-low BF gradient. This reverses to a low-to-high frequency gradient in the PAF. (B) Sample case of one representative NVHL rat.

For each rat, we constructed a map of the recording sites (10–15 tracks/mm^2^) that covered the A1 and PAF evenly and avoided blood vessels. Fig 3A and 3B show example maps of a sham and NVHL rat. The relative positions of the A1 and PAF were determined according to the characteristics of the BF (frequency of pure tone stimulus that evoked the maximum neural response) distribution across the AC (i.e., tonotopic gradient). There was a reversal of the BF gradients between the A1 and PAF. The BF in the A1 changed from low to high along the dorsoanterior direction, but this gradient changed to a dorsocaudal direction in the PAF (Fig 3A and 3B). In total, we sampled 603 sites in the left AC of 6 sham rats (A1, n = 194; PAF, n = 93) and 6 NVHL rats (A1, n = 224; PAF, n = 92). Within the 603 sites, we collected 486 LFPs responsive to the presented sound stimuli (sham rats: A1, n = 165; PAF, n = 69; NVHL rats: A1, n = 186; PAF, n = 66). The percentage of responsive sites in the A1 and PAF were similar between the sham and NVHL rats.

### Representative examples of LFPs in response to click trains in sham and NVHL rats

**Fig 4:**
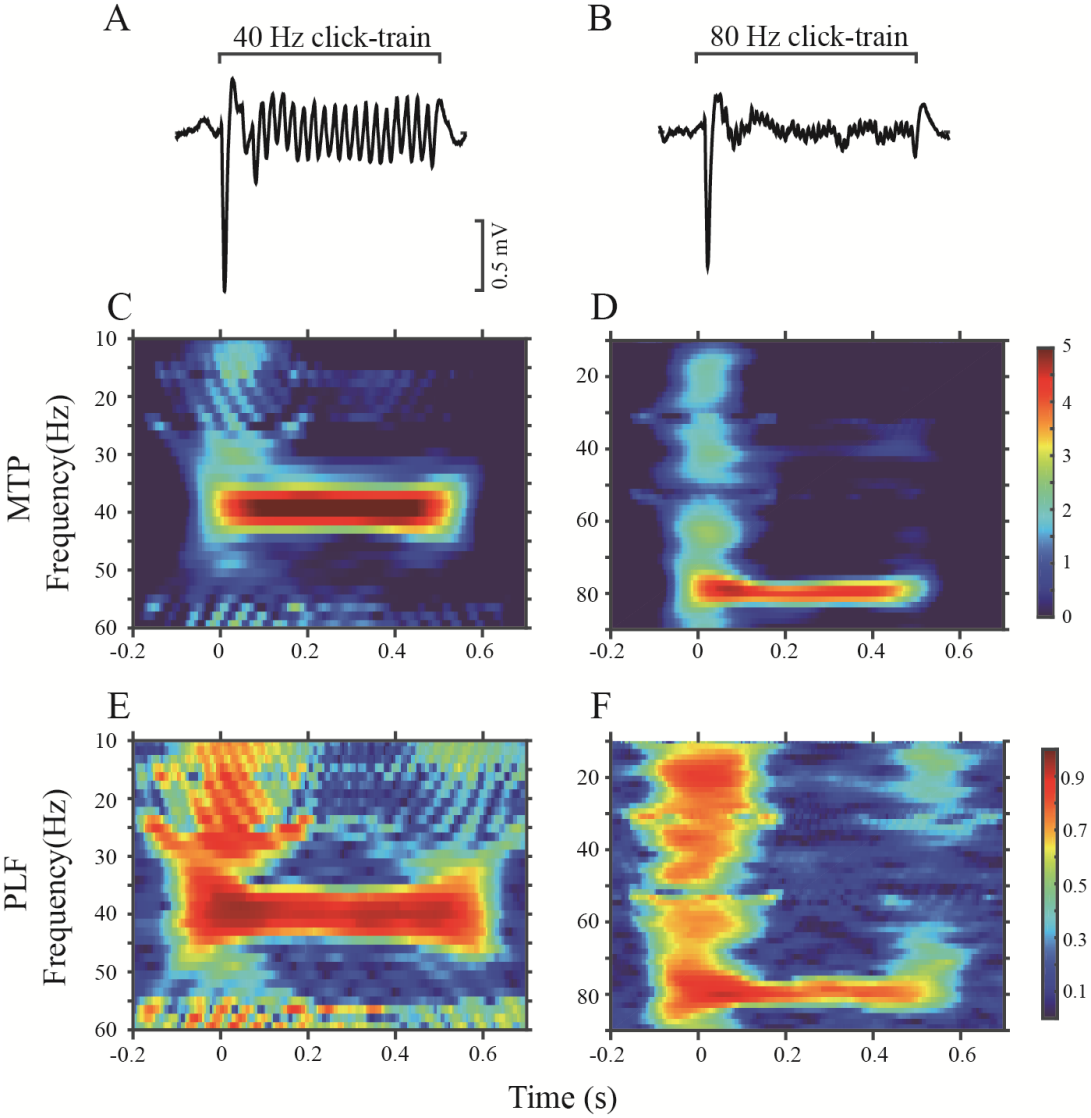
Example of the A1-ASSR in a representative sham rat. (A) and (B): averaged LFPs in response to 40- and 80-Hz click trains. (C) and (D): time-frequency plots of the MTP at 40- and 80-Hz stimulation. (E) and (F): time-frequency plots of the PLF at 40- and 80-Hz stimulation.

As shown by the representative LFP waves recorded in the A1 of sham rats (Fig 4A and 4B), the click trains of 40- and 80-Hz repetition rate could evoke a clear fluctuation in the LFP synchronization with the stimulation rhythm. For each individual trial of LFP, we computed the MTP and PLF to quantify the power and phase-locking of ASSR. The mean time—frequency plots for MTP and PLF, averaged over 60 trials of 40- and 80-Hz stimulation are shown in Fig 4C–4F. Based on this, it is evident that the LFP of this recording site shows a strong MTP and PLF at these stimulation frequencies (40 and 80 Hz).

**Fig 5:**
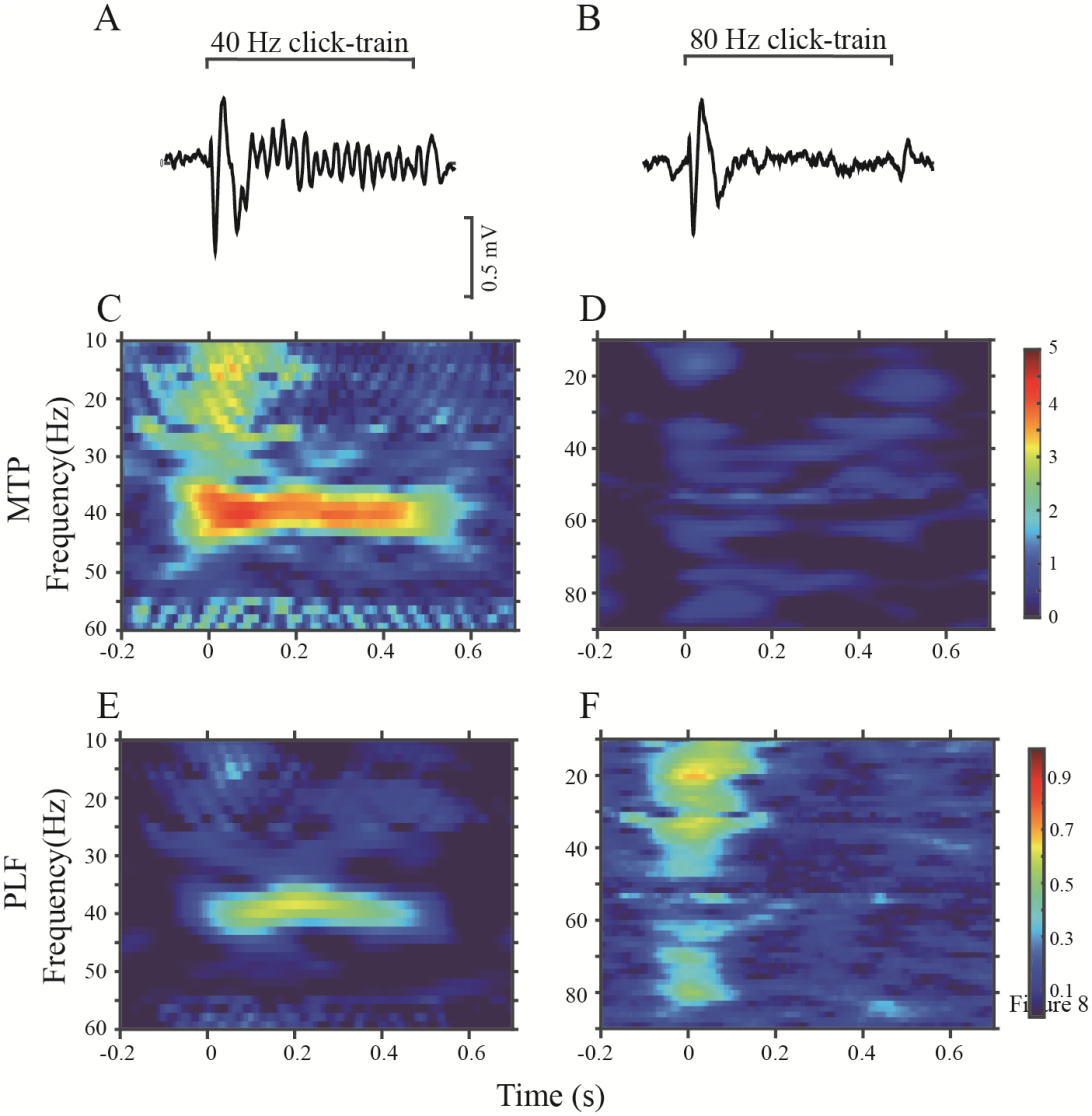
Example of the PAF-ASSR in a representative sham rat. The same format as Fig 4.

**Fig 6:**
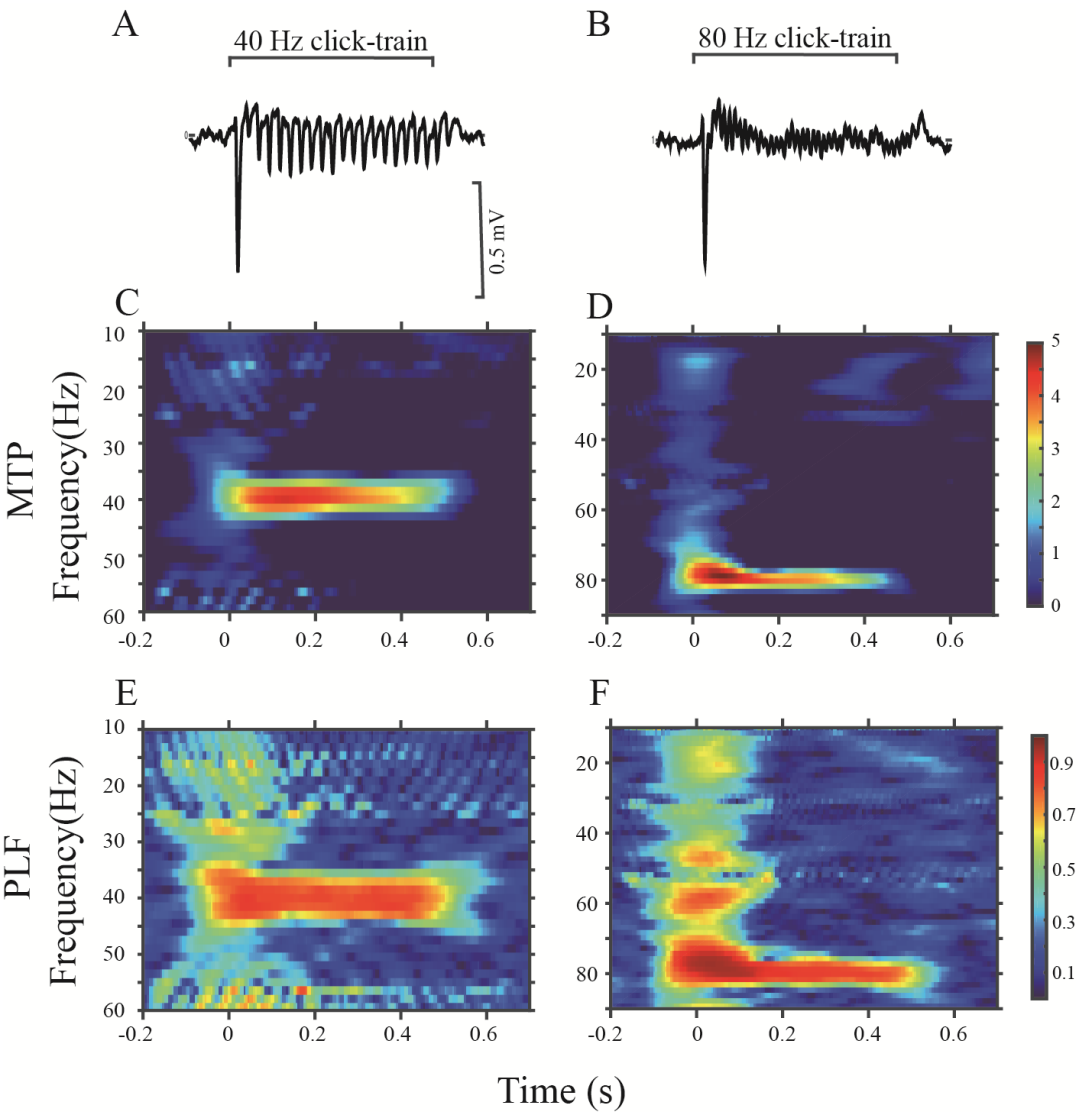
Example of the A1-ASSR in a representative NVHL rat. The same format as Fig 4.

**Fig 7:**
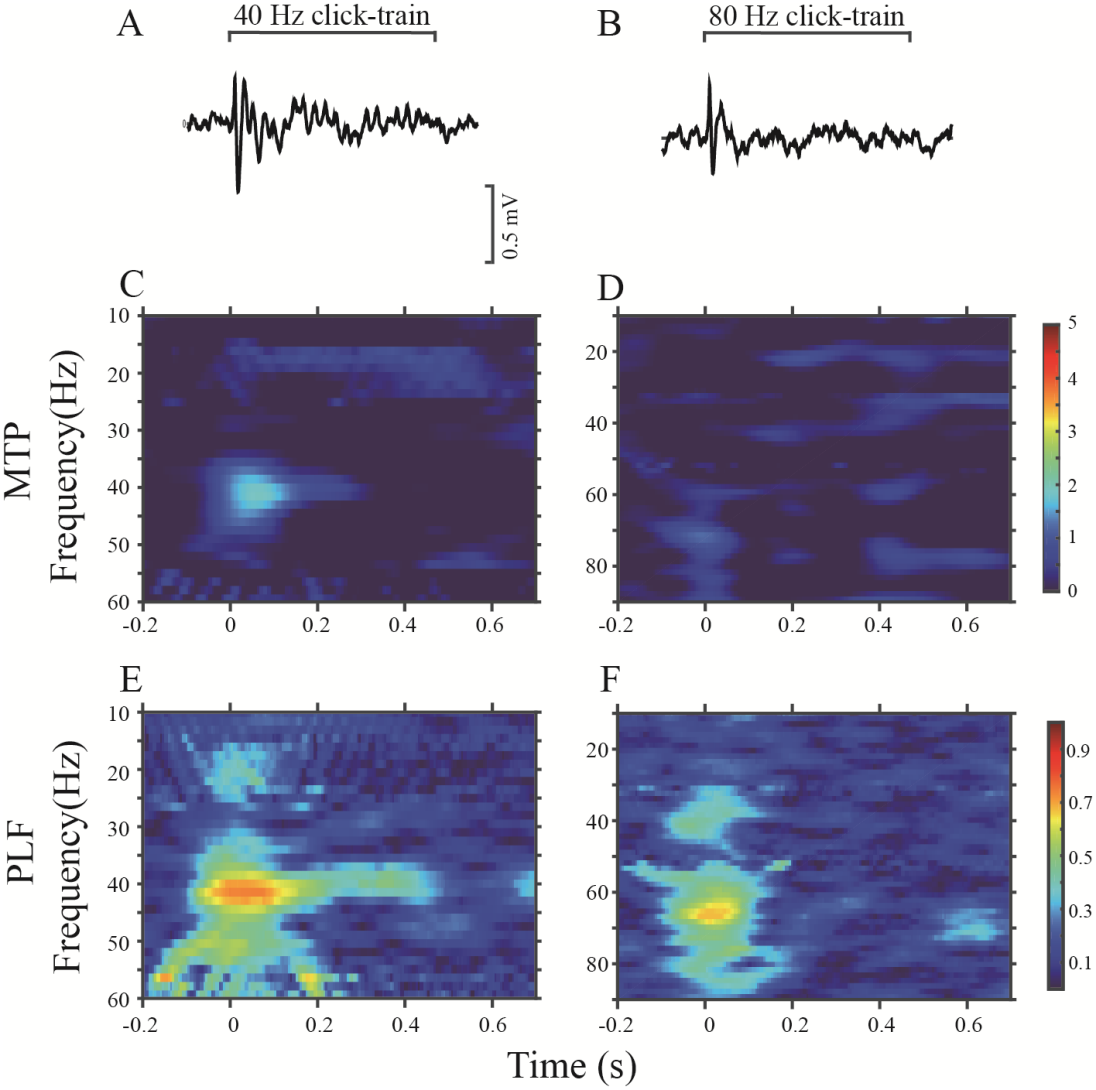
Example of the PAF-ASSR in a representative NVHL rat. The same format as Fig 4.

An example of an LFP recorded in the PAF is shown in Fig 5. Compared with the LFPs of the A1, the MTP and PLF of the PAF, particularly those elicited by the 80-Hz stimulation, were obviously decreased. Representative LFPs of NVHL rats are shown in Fig 6 (A1) and Fig 7 (PAF). Both 40- and 80-Hz click trains evoked robust ASSRs in the A1 of NVHL rats, but very weak in the PAF.

### Comparison of the population data of the MTP and PLF between sham and NVHL groups

**Fig 8:**
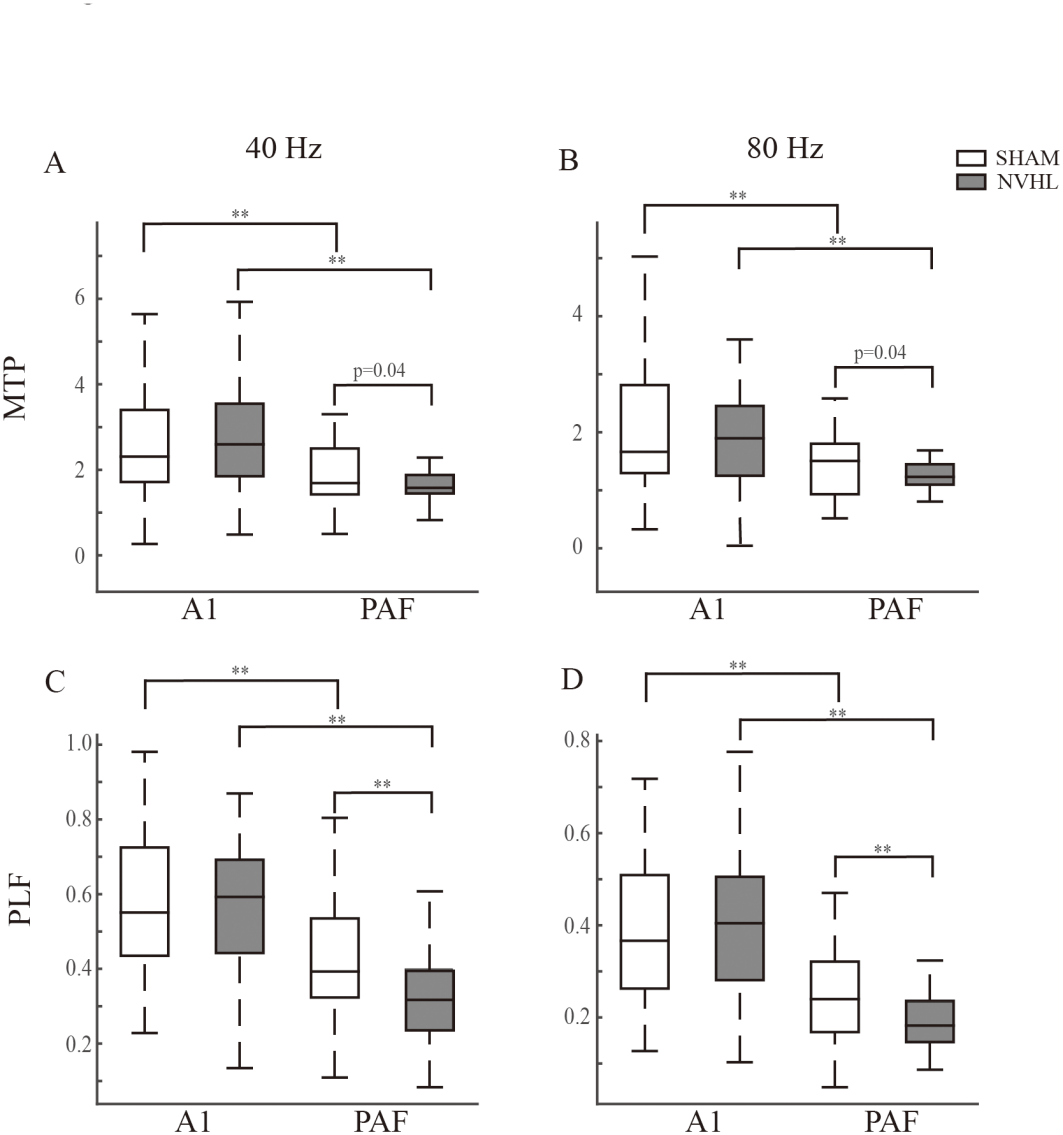
Comparison of the MTP and PLF in different AC regions of the NVHL and sham rats. (A) and (B) Boxplots of the MTP at 40- and 80-Hz stimulation. (C) and (D) Boxplots of the PLF at 40- and 80-Hz stimulation. The inner line and edges of the box represent the median, the 25, and 75 percentiles respectively, whiskers show the range as mean ± 3 SD. ** p < 0.01, * p < 0.05, *t* test.

Fig 8A shows the distribution of the 40-Hz MTP in the A1 and PAF for the sham and NVHL groups separately. Within the groups, the mean MTP of the A1 was significantly higher than that of the PAF (p < 0.01, *t* test; Cohen’s d effect size = 0.50 and 1.14). A comparison of the sham and NVHL groups revealed that the MTP was similar in the A1 (p = 0.82, *t* test; Cohen’s d effect size = 0.02), but significantly reduced in the PAF (p = 0.04, *t* test; Cohen’s d effect size = 0.50). The mean MTP evoked by the 80-Hz stimulation was generally smaller than that evoked by the 40-Hz one in both brain fields across the two groups (Fig 8B). A comparison of the 80-Hz MTPs between the different brain fields also revealed that the PAF had a lower MTP than the A1 in both sham and NVHL groups (p < 0.01, *t* test; Cohen’s d effect size = 0.48 and 0.95). In the NVHL rats, the 80-Hz MTP was reduced in the PAF (p = 0.04, *t* test; Cohen’s d effect size = 0.49), but not in the A1.

The distribution of the 40- and 80-Hz PLFs are shown in Fig 8C and 8D. Consistent with the results for the MTP, the A1 showed a stronger PLF than did the PAF, and the NVHL caused a significant reduction of the PLF in the PAF.

### Response duration of spike activity in sham and NVHL rats

**Fig 9:**
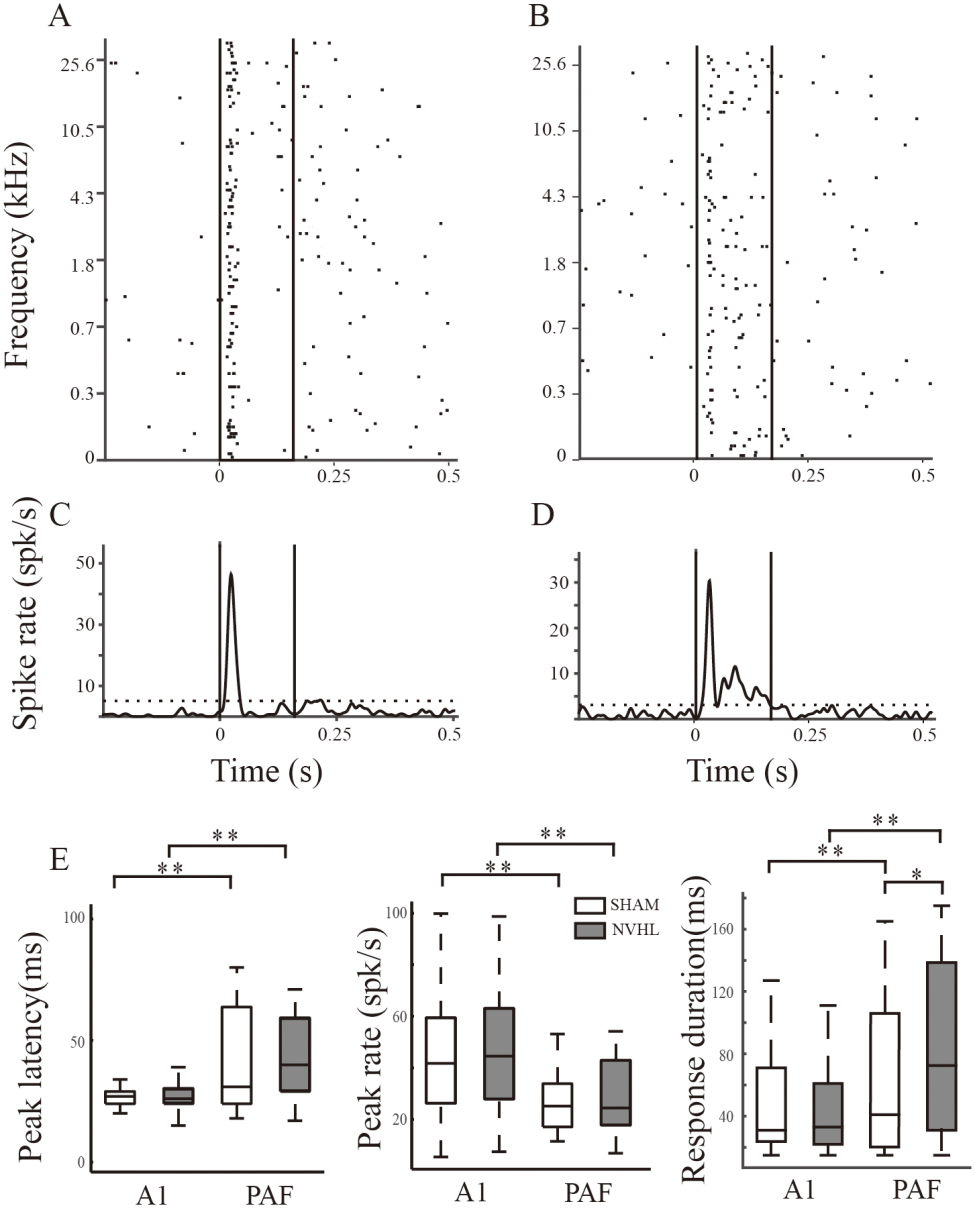
Response profiles of the spike activity evoked by pure tone stimuli. (A) and (B) Raster plot of the spike activity in response to pure tone stimuli at 125 different frequencies. One dot represents one spike. The two vertical lines mark the onset and cessation of the sound stimuli. (C) and (D) PSTH of the spike rate constructed from the raster plot. The two vertical lines mark the onset and cessation of the sound stimuli. The horizontal dotted line shows the threshold level (mean + 2 SD of the pre-stimulus background spike rate). (E) Boxplot of the response latency, rate, and duration in the different brain areas and groups. ** p < 0.01, * p < 0.05 *t* test.

We then investigated whether the reduction in ASSR, signified by the decreased synchronization of the neural response, is related to an alteration in the sound integration time (the duration of the neural response). At some recording sites, we could record extracellular spike activity from the same electrode while recording LFPs. In total, we collected 192 (134 A1, 58 PAF) and 176 (113 A1, 63 PAF) spike data for the sham and NVHL rats, respectively. We analyzed the spike responses to pure tone stimuli to estimate the duration of the neural response. Fig 9A and 9B show a representative example of spike activities recorded in the PAF of a sham and NVHL rat, respectively. PSTH was constructed in Fig 9C and 9D, from which an evoked response was identified using the threshold of mean + 2 SD of spontaneous spike rates. The latency and rate of the peak response and the response duration were estimated. Comparing the population data between the A1 and PAF neurons (Fig 9E), we found that the rate of peak responses in the A1 was significantly higher than that in the PAF, while the peak latency and response duration were shorter (p < 0.01, *t* test). This indicates that A1 neurons show a rapid and transient response to a single stimulus, therefore can follow repetitive stimuli more closely. This is consistent with the finding that ASSRs in the A1 were stronger than in the PAF. The response duration of PAF neurons was significantly longer in the NVHL than in the sham rats (p = 0.04, *t* test), while other parameters were similar between the two groups.

## Discussion

### Differences in ASSRs between the A1 and PAF

Most previous studies on recording ASSRs used brain surface EEG (8,22,23). In this study, we recorded LFPs from the cortex. The merit of intracranial over surface recordings is the fine spatial resolution. The disadvantage is that it is invasive, which limits its usage in humans. Using microelectrodes (tip diameter: <0.1 mm), we could isolate responses from the LFP and map them to specific cortical regions. The LFP recorded within the cortex is the averaged signal from coherent postsynaptic excitatory potentials generated by pyramidal neurons [27, 28]. It has been estimated that the spatial origin of LFPs is in the range of 1 mm [28]. In our investigation, we found that the MTP and PLF evoked by the 40- and 80-Hz click trains were both lower in the PAF than in the A1. Such a result may reflect the anatomic and functional differences between the two AC regions. The A1, the core region of the AC, receives thalamocortical projections from the lemniscal auditory thalamus. The PAF is a non-primary region and receives non-lemniscal projections from the auditory thalamus [29]. The cells in the lemniscal auditory thalamus have a narrow frequency tuning with short latencies and are tonotopically arranged, whereas those in the non-lemniscal thalamus are not tonotopically organized [30,31]. Consequently, PAF neurons show a broader frequency tuning, with longer and more variable latencies, and a more rapid habituation to repetitive stimuli, compared to A1 neurons [32–36]. The analysis of spike activity in this study also indicated that the response duration and latency of the PAF neurons was longer than that of the A1 neurons. We have previously examined the responses of single neurons to click trains in the AC of normal rats and found that compared to A1 neurons, PAF neurons had a lower capability to synchronize with the sound stimulation repetition rate [37]. Thus, the reduced ASSR in the PAF might be due to the decreased synchronization of the local PAF neural circuitry. This property signifies that, rather than representing the simple sound parameters, such as frequency or amplitude, with different discharge rates, PAF neurons could have a longer sound-encoding time and therefore integrate more acoustic information to generate selectivity for complex acoustic features, such as pitch and timbre [37–39].

One caveat that should be mentioned is that our electrophysiological data were recorded from anesthetized animals. Previous studies on both human and animal subjects have shown that the ASSR is attenuated by anesthesia [40–44]. However, given that our data from the A1 and PAF were collected under the same anesthesia conditions, their comparison is still meaningful. Nevertheless, caution should be taken when interpreting these results, as we cannot completely exclude the possibility that the ASSRs of the A1 and PAF neurons are differently attenuated by the anesthesia.

### PAF dysfunction in NVHL rats

Our results showed that the PAF-ASSR was significantly reduced in NVHL rats, while the A1-ASSR was less affected. This result suggests that NVHL mainly disrupts the neural network operating in the non-primary AC. Anatomic studies have revealed that non-primary fields of the AC receive projections from both the non-lemniscal medial geniculate body [36] and the primary AC [45]. Non-primary AC outputs project to limbic and prefrontal brain areas, which are involved in attention, motivation and emotion [46, 47]. Thus, the non-primary fields of the AC could play an important role in the integration of auditory inputs. The abnormalities of the ASSR observed in the PAF indicate that auditory processing integration might be impaired in the NVHL model. However, the representational functions undertaken by the A1 remain intact. This possibility is supported by our result that, in NVHL rats, the response duration of the PAF neurons was extended, but remained unchanged for the A1 neurons.

Some histopathological changes caused by the NVHL might contribute to the observed effects on the ASSR. Histological studies have reported that the number of cortical neurons was reduced in NVHL rats [16, 20, 48]. This might result from a disturbed thalamocortical innervation [18,49,50]. Though no direct connection between the hippocampus and the AC has been reported yet, indirect connections may exit via the circuits between the hippocampus, the amygdala, and the auditory thalamus [17]. On the other hand, the medial auditory thalamus is adjacent to the lesioned area of the hippocampus [29], which could be destroyed by ibotenate. It has been found that NVHL rats show calcium deposits in the medial auditory thalamus [18,51]. Moreover, this calcification could also be caused by glial damage during the neonatal period [52]. These histopathological changes might lead to inappropriate connectivity within the thalamocortical pathways, particularly the ones involving the PAF. This possibility needs to be investigated in the future by combining histological and electrophysiological methods.

## Conclusion

We found that the NVHL rat model exhibits a significantly reduced ASSR, a common phenomenon observed in electrophysiological examinations of schizophrenia patients. EEG measurements have consistently shown reductions in the power and phase-locking of ASSRs in schizophrenia patients, particularly in the gamma band (30–100 Hz) [4–8]. A previous study with NVHL rats did not find an obvious deficit in the 40-Hz ASSR [22]. In the present study, we recorded LFPs in the AC of NVHL rats and found that the MTP and PLF of both the 40- and 80-Hz ASSRs were reduced in the PAF. Our results provide robust evidence for consistency between ASSRs recorded via an intracortical microelectrode and those recorded by scalp EEG, thus bridging the gap between electrophysiological studies of animals and clinical examinations of schizophrenia patients.

## Abbreviations

A1: primary auditory cortex
AC: auditory cortex
ASSR: auditory steady state response
LFP: local field potential
MTP: Mean trial power
NVHL: neonatal ventral hippocampal lesion
PAF: posterior auditory field
PLF: phase-locking factor
